# 1,4-Bis(2-pyridylimino­meth­yl)benzene

**DOI:** 10.1107/S1600536809039853

**Published:** 2009-10-07

**Authors:** Li-Hua Huo, Shan Gao, Seik Weng Ng

**Affiliations:** a College of Chemistry and Materials Science, Heilongjiang University, Harbin 150080, People’s Republic of China; b Department of Chemistry, University of Malaya, 50603 Kuala Lumpur, Malaysia

## Abstract

In the crystal structure of the title compound, C_18_H_14_N_4_, the mol­ecule assumes 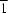 site symmetry with the centroid of the benzene ring located on the inversion center. The mol­ecule is almost flat, with a dihedral angle of 2.70 (9)° between the pyridine and benzene rings.

## Related literature

For the synthesis, see: D’Alelio *et al.* (1967 ▶). Terephthaldehyde condenses directly with 2-amino­pyridine to form 4-(bis­(2-pyridylamino)meth­yl)benzaldehyde; see: Zhu *et al.* (2003 ▶).
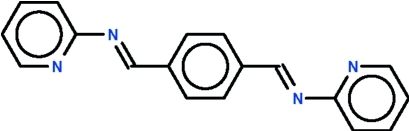


## Experimental

### 

#### Crystal data

C_18_H_14_N_4_
*M* *_r_* = 286.33
Monoclinic, 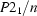

*a* = 6.1579 (8) Å
*b* = 18.911 (3) Å
*c* = 6.5956 (11) Å
β = 109.746 (5)°
*V* = 722.90 (18) Å^3^
*Z* = 2
Mo *K*α radiation
μ = 0.08 mm^−1^
*T* = 293 K
0.30 × 0.26 × 0.21 mm

#### Data collection

Rigaku R-AXIS RAPID IP diffractometer
Absorption correction: multi-scan (*ABSCOR*; Higashi, 1995 ▶) *T*_min_ = 0.976, *T*_max_ = 0.983
7013 measured reflections
1657 independent reflections
886 reflections with *I* > 2σ(*I*)
*R*_int_ = 0.048

#### Refinement

*R*[*F* ^2^ > 2σ(*F* ^2^)] = 0.048
*wR*(*F* ^2^) = 0.168
*S* = 1.05
1657 reflections
100 parameters
H-atom parameters constrained
Δρ_max_ = 0.15 e Å^−3^
Δρ_min_ = −0.21 e Å^−3^

### 

Data collection: *RAPID-AUTO* (Rigaku, 1998 ▶); cell refinement: *RAPID-AUTO*; data reduction: *CrystalClear* (Rigaku/MSC, 2002 ▶); program(s) used to solve structure: *SHELXS97* (Sheldrick, 2008 ▶); program(s) used to refine structure: *SHELXL97* (Sheldrick, 2008 ▶); molecular graphics: *X-SEED* (Barbour, 2001 ▶); software used to prepare material for publication: *publCIF* (Westrip, 2009 ▶).

## Supplementary Material

Crystal structure: contains datablocks global, I. DOI: 10.1107/S1600536809039853/xu2625sup1.cif

Structure factors: contains datablocks I. DOI: 10.1107/S1600536809039853/xu2625Isup2.hkl

Additional supplementary materials:  crystallographic information; 3D view; checkCIF report

## Figures and Tables

**Fig. 1.**
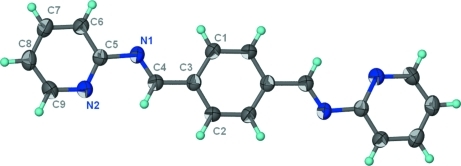
Thermal ellipsoid plot (Barbour, 2001) of the title compound at the 50% probability level; hydrogen atoms are drawn as spheres of arbitrary radius.

## supplementary crystallographic information

### Experimental

To a solution of terephthaladehyde (1 mmol) in methanol was added a solution of 2-aminopyridine (2 mmol) and cobalt acetate trihydrate (1 mmol) in methanol. The mixture was heated to 333 K for one hour. The pale yellow solution was filtered. Colorless crystals were isolated from the filtrate after several days. CH&N elemental analysis. Calc. for C_18_H_14_N_4_: C 75.51, H 4.93, N 19.57%; found: C 75.53, H 4.98, N 19.53%.

### Refinement

Carbon-bound H-atoms were placed in calculated positions (C—H 0.93 Å) and were included in the refinement in the riding model approximation, with *U*(H) set to 1.2*U*(C).

### Crystal data

**Table:**

C_18_H_14_N_4_	*F*(000) = 300
*M**_r_* = 286.33	*D*_x_ = 1.315 Mg m^−^^3^
Monoclinic, *P*2_1_/*n*	Mo *K*α radiation, λ = 0.71073 Å
Hall symbol: -P 2yn	Cell parameters from 3577 reflections
*a* = 6.1579 (8) Å	θ = 3.5–27.5°
*b* = 18.911 (3) Å	µ = 0.08 mm^−^^1^
*c* = 6.5956 (11) Å	*T* = 293 K
β = 109.746 (5)°	Prism, colorless
*V* = 722.90 (18) Å^3^	0.30 × 0.26 × 0.21 mm
*Z* = 2	

### Data collection

**Table:**

Rigaku R-AXIS RAPID IP diffractometer	1657 independent reflections
Radiation source: fine-focus sealed tube	886 reflections with *I* > 2σ(*I*)
graphite	*R*_int_ = 0.048
ω scans	θ_max_ = 27.5°, θ_min_ = 3.5°
Absorption correction: multi-scan (*ABSCOR*; Higashi, 1995)	*h* = −7→7
*T*_min_ = 0.976, *T*_max_ = 0.983	*k* = −24→24
7013 measured reflections	*l* = −8→8

### Refinement

**Table:**

Refinement on *F*^2^	Primary atom site location: structure-invariant direct methods
Least-squares matrix: full	Secondary atom site location: difference Fourier map
*R*[*F*^2^ > 2σ(*F*^2^)] = 0.048	Hydrogen site location: inferred from neighbouring sites
*wR*(*F*^2^) = 0.168	H-atom parameters constrained
*S* = 1.05	*w* = 1/[σ^2^(*F*_o_^2^) + (0.0811*P*)^2^ + 0.0419*P*] where *P* = (*F*_o_^2^ + 2*F*_c_^2^)/3
1657 reflections	(Δ/σ)_max_ = 0.001
100 parameters	Δρ_max_ = 0.15 e Å^−^^3^
0 restraints	Δρ_min_ = −0.21 e Å^−^^3^

### Fractional atomic coordinates and isotropic or equivalent isotropic displacement parameters (Å^2^)

**Table:**

	*x*	*y*	*z*	*U*_iso_*/*U*_eq_	
N1	0.2738 (3)	0.57623 (8)	0.4070 (3)	0.0484 (5)	
N2	0.4005 (3)	0.67692 (8)	0.6348 (3)	0.0547 (5)	
C1	0.3212 (3)	0.48484 (9)	0.0748 (3)	0.0490 (5)	
H1	0.2001	0.4745	0.1242	0.059*	
C2	0.6436 (3)	0.55629 (10)	0.0822 (3)	0.0501 (6)	
H2	0.7408	0.5945	0.1371	0.060*	
C3	0.4653 (3)	0.54201 (9)	0.1610 (3)	0.0422 (5)	
C4	0.4303 (3)	0.58674 (9)	0.3268 (3)	0.0470 (5)	
H4	0.5285	0.6251	0.3762	0.056*	
C5	0.2518 (3)	0.62320 (9)	0.5657 (3)	0.0427 (5)	
C6	0.0719 (4)	0.61176 (10)	0.6427 (3)	0.0505 (6)	
H6	−0.0267	0.5735	0.5940	0.061*	
C7	0.0405 (4)	0.65755 (10)	0.7920 (3)	0.0566 (6)	
H7	−0.0817	0.6513	0.8431	0.068*	
C8	0.1919 (4)	0.71275 (11)	0.8649 (4)	0.0585 (6)	
H8	0.1762	0.7443	0.9673	0.070*	
C9	0.3676 (4)	0.71989 (10)	0.7815 (4)	0.0598 (6)	
H9	0.4701	0.7572	0.8310	0.072*	

### Atomic displacement parameters (Å^2^)

**Table:**

	*U*^11^	*U*^22^	*U*^33^	*U*^12^	*U*^13^	*U*^23^
N1	0.0544 (10)	0.0491 (9)	0.0460 (11)	−0.0014 (8)	0.0225 (9)	−0.0062 (7)
N2	0.0572 (11)	0.0523 (10)	0.0595 (12)	−0.0048 (8)	0.0260 (9)	−0.0111 (8)
C1	0.0530 (12)	0.0510 (11)	0.0503 (13)	−0.0033 (9)	0.0268 (10)	−0.0034 (9)
C2	0.0523 (12)	0.0492 (11)	0.0540 (13)	−0.0120 (9)	0.0250 (11)	−0.0078 (9)
C3	0.0472 (11)	0.0413 (10)	0.0389 (11)	0.0030 (8)	0.0158 (9)	0.0011 (8)
C4	0.0505 (12)	0.0453 (11)	0.0478 (13)	−0.0031 (9)	0.0202 (10)	−0.0036 (9)
C5	0.0452 (11)	0.0419 (10)	0.0415 (11)	0.0036 (8)	0.0156 (9)	0.0002 (8)
C6	0.0544 (13)	0.0496 (11)	0.0531 (14)	−0.0030 (9)	0.0256 (11)	−0.0022 (9)
C7	0.0674 (14)	0.0548 (12)	0.0588 (14)	0.0064 (10)	0.0361 (12)	0.0009 (10)
C8	0.0741 (14)	0.0513 (12)	0.0564 (14)	0.0079 (11)	0.0305 (12)	−0.0071 (10)
C9	0.0673 (14)	0.0500 (11)	0.0639 (15)	−0.0090 (10)	0.0247 (12)	−0.0159 (10)

### Geometric parameters (Å, °)

**Table:**

N1—C4	1.262 (2)	C3—C4	1.455 (3)
N1—C5	1.414 (2)	C4—H4	0.9300
N2—C9	1.330 (2)	C5—C6	1.383 (3)
N2—C5	1.340 (2)	C6—C7	1.373 (3)
C1—C2^i^	1.369 (3)	C6—H6	0.9300
C1—C3	1.392 (3)	C7—C8	1.374 (3)
C1—H1	0.9300	C7—H7	0.9300
C2—C1^i^	1.369 (3)	C8—C9	1.376 (3)
C2—C3	1.391 (3)	C8—H8	0.9300
C2—H2	0.9300	C9—H9	0.9300
			
C4—N1—C5	119.30 (17)	N2—C5—N1	120.16 (16)
C9—N2—C5	117.11 (17)	C6—C5—N1	117.58 (17)
C2^i^—C1—C3	120.54 (17)	C7—C6—C5	119.28 (19)
C2^i^—C1—H1	119.7	C7—C6—H6	120.4
C3—C1—H1	119.7	C5—C6—H6	120.4
C1^i^—C2—C3	121.24 (17)	C6—C7—C8	119.14 (19)
C1^i^—C2—H2	119.4	C6—C7—H7	120.4
C3—C2—H2	119.4	C8—C7—H7	120.4
C2—C3—C1	118.21 (17)	C7—C8—C9	117.83 (19)
C2—C3—C4	120.26 (17)	C7—C8—H8	121.1
C1—C3—C4	121.52 (16)	C9—C8—H8	121.1
N1—C4—C3	123.46 (18)	N2—C9—C8	124.36 (19)
N1—C4—H4	118.3	N2—C9—H9	117.8
C3—C4—H4	118.3	C8—C9—H9	117.8
N2—C5—C6	122.26 (17)		
			
C1^i^—C2—C3—C1	−0.5 (3)	C4—N1—C5—N2	−2.1 (3)
C1^i^—C2—C3—C4	−179.58 (18)	C4—N1—C5—C6	177.18 (19)
C2^i^—C1—C3—C2	0.5 (3)	N2—C5—C6—C7	1.3 (3)
C2^i^—C1—C3—C4	179.57 (18)	N1—C5—C6—C7	−177.98 (17)
C5—N1—C4—C3	−179.24 (17)	C5—C6—C7—C8	−1.5 (3)
C2—C3—C4—N1	−179.11 (19)	C6—C7—C8—C9	0.8 (3)
C1—C3—C4—N1	1.9 (3)	C5—N2—C9—C8	−0.2 (3)
C9—N2—C5—C6	−0.5 (3)	C7—C8—C9—N2	0.1 (4)
C9—N2—C5—N1	178.83 (18)		

Symmetry codes: (i) −*x*+1, −*y*+1, −*z*.
